# The insight of *in vitro* and *in silico* studies on cholinesterase inhibitors from the roots of *Cimicifuga dahurica* (Turcz.) Maxim.

**DOI:** 10.1080/14756366.2018.1491847

**Published:** 2018-10-05

**Authors:** Jang Hoon Kim, Nguyen Phuong Thao, Yoo Kyong Han, Young Suk Lee, Bui Thi Thuy Luyen, Ha Van Oanh, Young Ho Kim, Seo Young Yang

**Affiliations:** a Radiation Breeding Research Center, Advanced Radiation Technology Institute, Korea Atomic Energy Research Institute, Jeoungeup, Jeollabuk-do, Republic of Korea;; b Institute of Marine Biochemistry (IMBC), Vietnam Academy of Science and Technology (VAST), Hanoi, Vietnam;; c College of Pharmacy, Chungnam National University, Daejeon, Republic of Korea;; d Department of Pharmaceutical Industry, Hanoi University of Pharmacy, Hanoi, Vietnam

**Keywords:** *Cimicifuga dahurica*, Ranunculaceae, cholinestereases inhibitor, molecular simulation

## Abstract

Cholinesterases (ChEs) are enzymes that break down neurotransmitters associated with cognitive function and memory. We isolated cinnamic acids (**1** and **2**), indolinones (**3** and **4**), and cycloartane triterpenoid derivatives (**5**–**19**) from the roots of *Cimicifuga dahurica* (Turcz.) Maxim. by chromatography. These compounds were evaluated for their inhibitory activity toward ChEs. Compound **1** was determined to have an IC_50_ value of 16.7 ± 1.9 μM, and to act as a competitive inhibitor of acetylcholinesterase (AChE). Compounds **3**, **4** and **14** were found to be noncompetitive with IC_50_ values of 13.8 ± 1.5 and 6.5 ± 2.5 μM, and competitive with an IC_50_ value of 22.6 ± 0.4 μM, respectively, against butyrylcholinesterase (BuChE). Our molecular simulation suggested each key amino acid, Tyr337 of AChE and Asn228 of BuChE, which were corresponded with potential inhibitors **1**, and **3** and **4**, respectively. Compounds **1** and **4** were revealed to be promising compounds for inhibition of AChEs and BuChEs, respectively.

## Introduction

Cholinesterases (ChEs), which are enzymes that hydrolyse choline esters, are classified as acetylcholinesterase (EC 3.1.1.7, AChE) and butyrylcholinesterase (EC 3.1.1.8, BuChE)^1^. AChE is responsible for the conversion of acetylcholine (ACh) into choline and acetic acid in cholinergic synapses. AChE is formed as a tetramer of ∼70-kDa monomeric subunits^1^^,^^2^. Its 3D structure was revealed by examining the enzyme of electric eels^3^. AChE has an active site with α-helix and β-sheet structures and a catalytic triad of serine, histidine, and glutamic acid^1^^,^^3^. BuChE, an enzyme that breaks down artificial butyrylcholine, is known to hydrolyse ACh and other ester derivatives in the body^4^^,^^5^. BuChE, which is a tetrameric serine esterase consisting of monomers of ∼90-kDa molecular mass, showed over 65% structural similarity to AChE^4^^,^^6^. ACh is a neurotransmitter that is produced from the acetylation reaction of choline and acetyl-CoA by choline acetyltransferase, and is distributed in the central and peripheral nervous systems^7^. ACh plays a key role in nerve-nerve communication by binding to ACh receptors^8^. This molecule is associated with maintenance of cognitive function and memory^5^^,^^8^. In particular, Alzheimer’s disease (AD) patients are characterised by a decline in ACh levels^8^. Two ChEs have been regarded as target enzymes for treatment of AD^1^^,^^5^^,^^8^.

*Cimicifuga dahurica* (Turcz.) Maxim., in the family Ranunculaceae, is commonly called “*shengma*” and is distributed throughout Korea, Japan, China and Russia^9^. In China, the rhizomes of *C. dahurica* have been used as a traditional medicine to treat headaches and toothaches^10^. Phytochemical studies of this plant indicated the presence of cycloartane triterpenoids and cinnamic acid derivatives^9^. These compounds exhibit neuroprotective activity and enhance cell viability by eliminating H_2_O_2_ in PC12 cells^9^^,^^11^. Cycloartane triterpenoids have anti-tumour activities including induction of apoptosis and G_2_/M cell cycle arrest in solid tumours, blood tumours and drug-resistant tumours^10^.

These findings led us to search for products that block the catalytic reaction of ChE. We isolated compounds **1**–**19** from the roots of *C. dahurica* using open column chromatography (CC). These compounds were tested for interactions with both AChE and BuChE *in vitro*. Through molecular simulation, the inhibitor- ChE complex structure was predicted visually using the Autodock 4.2 programme. The complex that was constructed considered the interaction between the inhibitor and ChE in terms of molecular dynamics (MDs).

## Materials and methods

### General experimental procedures

Optical rotations were measured using a JASCO P-2000 polarimeter (JASCO, Oklahoma, OK, USA). IR spectra were obtained on a Bruker TENSOR 37 FT-IR spectrometer (Bruker, Billerica, MA, USA). NMR spectra were recorded on JEOL JNM-AL 400 MHz and JEOL ECA 600 MHz spectrometer (JEOL, Peabody, MA, USA), chemical shift (*δ*) are expressed in ppm with reference to the TMS signals. Gas chromatography spectra were recorded on a Shimadzu-2010 spectrometer (Shimadzu, Kyoto, Japan), SPB-1 capillary (30 m × 0.25 mm and 30 m × 0.32 mm); Mightysil RP-18 GP, Kanto Chemical, 10 × 250 mm. The electrospray ionisation and the high-resolution electrospray ionisation mass spectrometer were operated in the positive-ion mode, with sodium iodide being used for mass calibration from an Agilent 6530 Accurate-Mass Q-TOF LC/MS system (Micromass, Wythenshawe, UK). CC was conducted using on 65–250 or 230–400 mesh silica gel (Sorbent Technologies, Atlanta, GA, USA), porous polymer gel (Diaion® HP-20, 20–60 mesh, Mitsubishi Chemical, Tokyo, Japan), Sephadex™ LH-20 (Supelco, Bellefonte, PA, USA), octadecyl silica (ODS, 50 *μ*m, Cosmosil 140 C_18_-OPN, Nacalai Tesque), and YMC RP-C_18_ resins (30–50 *μ*m, Fuji Silysia Chemical). Analytical thin layer chromatography (TLC) systems were performed on precoated silica gel 60 F_254_ (1.05554.0001, Merck) and RP-18 F_254S_ plates (1.15685.0001, Merck) and compounds were visualised by spraying with 10% H_2_SO_4_ in water and then heating for 1.5–2 min. All procedures were carried out with solvents purchased from commercial sources that were used without further purification.

### Chemicals and reagents

AChE (C3389), acetylthiocholine iodide (A5751), BuChE (C1057), butyrylthiocholine iodide (B3253) and 5,5-dithiobis(2-nitrobenzoic acid) (DTNB) were purchased from Sigma-Aldrich (St Louis, MO, USA).

### Plant material

The roots (3.5-years old) of *C. dahurica* were purchased from a herbal company, Naemome Dah, Ulsan, Korea, in February 2016. This sample was identified by Prof. Y.H. Kim. A voucher specimen (CNU-16003) representing this collection has been deposited at the Herbarium of the College of Pharmacy, Chungnam National University, Daejeon, Korea.

### Extraction and isolation

The roots of *C. dahurica* (2.5 kg) were extracted three times with 5.0 L of 95% ethanol at 40 °C. Concentrated ethanol extract (65.3 g) was suspended in distilled water and progressively fractioned with *n*-hexane (9.6 g), dichloromethane (15.2 g) and water (40.5 g) fractions.

Dichloromethane fraction was subjected to silica gel CC by using gradient solvent system of *n*-hexane and EtOAc (from 95:5 to 2.5:5) to achieve seven fractions (D1?D7). D3 fraction was chromatographed by Sephadex LH-20 CC with MeOH-H_2_O solvent (95:5) to isolate compounds **7** (10.2 mg) and **10** (8.9 mg). Compounds **5** (13.5 mg), **6** (4.0 mg), **9** (3.8 mg) and **13** (3.1 mg) were purified by silica gel CC with an isocratic solvent system of *n*-hexane and EtOAc (2:1) from D4 fraction. D5 fraction was chromatographed by RP-C_18_CC using mixture solvent system of acetone and H_2_O (1:4) to afford compounds **1** (3 mg), **8** (5.6 mg) and **12** (3.0 mg). D7 fraction was purified by over Sephadex LH-20 CC with MeOH solvent to give three fractions (D7.1?D7.3). D7.1 fraction was subjected to RP-C_18_ CC by using solvent system of MeOH and H_2_O (5.5:1) to obtain compounds **3** (20 mg) and **4** (5 mg). Compounds **2** (32.6 mg) and **11** (5 mg) were isolated by over silica gel CC with solvent system of CH_2_Cl_2_ and acetone (4:1) from D7 fraction. H_2_O fraction was subjected to a Diaion HP-20 CC by using gradient solvent system of MeOH and H_2_O (from 25:75 to 100:0) to give four fractions (H1–H4). H2 fraction was isolated as compounds **14** (11.7 mg), **15** (18.2 mg), **18** (15.2 mg) and **19** (10.1 mg) by RP-C_18_ CC with gradient solvent system of MeOH and H_2_O (from 35:65 to 100:0). H4 fraction was purified by over RP-C_18_ CC with solvent system of MeOH and H_2_O (35:65) to achieve compounds **16** (10.3 mg) and **17** (3.5 mg).

### ChE assay

AChE and BuChE inhibition assays were performed as described by Othman *et al*.^12^ with some modifications. Briefly, each 130 μL of AChE (∼0.05 U/mL) and BuChE (∼0.05 U/mL) in 50 mM phosphate buffer (pH 7.4) was added to 96-well plates containing 20 μL of MeOH or sample dissolved in MeOH. 25 μL acetylthiocholine iodide (5 mM) or butyrylthiocholine iodide (5 mM), and 25 μL DTNB (1 mM) were added into the mixture in order. After initiating ChE reaction at 37 °C, the products were scanned at 475 nm UV-Vis photometer for 20 min. The inhibition activity was calculated using the following equation:

Inhibitory activity (%)=[(Δcontrol−Δsample)/Δcontrol]×100.

Where control and sample were the intensity of control and inhibitor after 20 min, respectively.

The ChE inhibitory activity of each sample was expressed as IC_50_ (µM required to inhibit the hydrolysis of the ChE substrates by 50%) determined from the log-dose inhibition curve.

### Molecular docking of inhibitor with ChE

Molecular docking was performed as previously described using the Autodock 4.2 programme (La Jolla, CA, USA)^12^. Single bond of ligand was flexibly assigned by using torsion tree of Autodocktools. Each pdb files of AChE (pdb ID: 1C2B) and BuChE (pdb ID: 4BDS) were downloaded from RCSB protein data bank. Achieved protein was added in hydrogens, and then this was assigned with compute gasteiger charges. For the docking, the grid containing activity site or all protein was set. Ligand was docked into that with default values of genetic algorithm parameters (number of GA runs: 50, maximun number of evals: 25,000,000). The result was presented with Ligplot (Cambridge, UK) and Chimaera (San Francisco, CA, USA).

### Molecular simulation of inhibitor with ChE

MDs were performed to simulate the complex of ligand with protein by the Gromacs version 4.6.5 package. Itp and gro files of ligand were built at Prodrg server. Gro and topology files of ChE were generated by pdbgmx utility. These were edited to add ligand files. The ligand with protein was dissolved in water molecules of a cubic box with a size of 12 × 12 × 12 containing six sodium ions (1.0 Å distance). Moreover, then this complex was minimised until it reach the maximal force of 10 kJ/mol. Each NVT and NPT was simulated at 300 K temperature and 1 bar pressure for 100 ps in the order, respectively. Lastly, equilibrated complex was subjected to MD simulation for 10,000 ps.

### Statistical analysis

Statistical significance was determined using a one-way analysis of variance and Students t-test (Systat Inc., Evanston, IL, USA). A *p* value <0.01 was considered significant. All results are presented as the mean ± SEM.

## Results and discussion

### Isolation and identification

**Figure 1.**
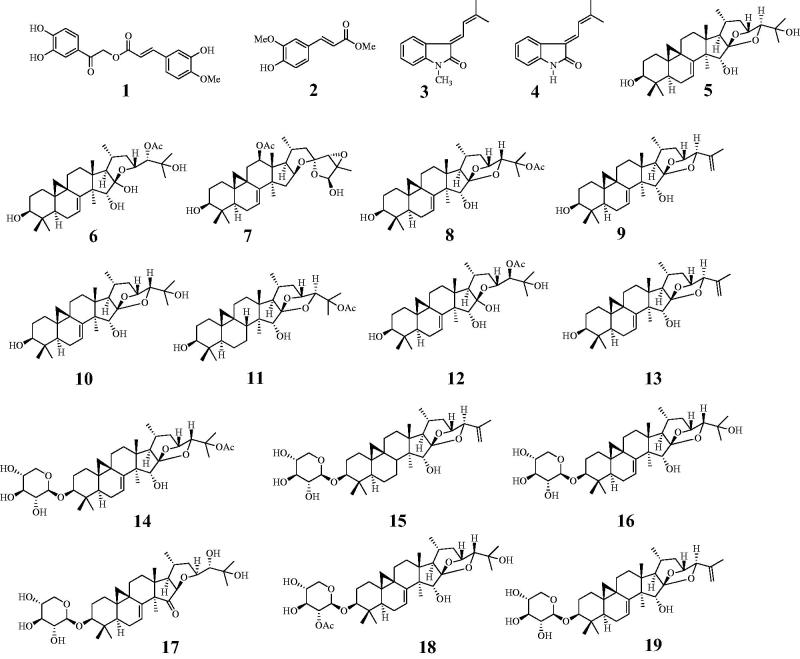
Structures of isolated compounds **1**–**19** from *C. dahurica*.

An ethanol extract of the roots of *C. dahurica* was progressively partitioned into *n*-hexane, dichloromethane, and water fractions. The dichloromethane and water fractions were subjected to various CC methods to obtain compounds **1**–**13** and **14**–**19**, respectively. These compounds were investigated based on spectroscopic data and comparison with previous reports. The nineteen extracted compounds were identified as cimiciphenone (**1**)^13^, ferulic acid methyl ester (**2**)^13^, (*E*)-3‐(3′-methyl-2′-butenylidene)-1-methyl-2-indolinone (**3**)^13^, (*E*)-3‐(3′-methyl-2′-butenylidene)-2-indolinone (**4**)^13^, 7,8-didehydrocimigenol (**5**), 24-*epi*-24-*O*-acetyl-7,8-didehydroshengmanol (**6**)^14^, 25-triepoxy-12*β*-acetoxy-3*β*,26-dihydroxy-9,19-cyclolanost-7-ene (**7**)^15^, 25-*O*-acetyl-7,8-didehydrocimigenol (**8**)^16^, 25-anhydro-7,8-didehydrocimigenol (**9**)^15^, 24-*epi*-7,8-didehydrocimigenol (**10**)^16^, 25-*O*-acetylcimigenol (**11**)^15^, 24-*epi*-24-*O*-acetyl-7,8-didehydroshengmanol (**12**)^14^, 25-anhydrocimigenol (**13**)^15^, 25-*O*-acetyl-7,8-didehydrocimigenol 3-*O*-*β*-D-xylopyranoside (**14**)^14^, 25-anhydrocimigenol-3-*O*-*β*-D-xylopyranoside (**15**)^17^, 24-*epi*-7,8-didehydrocimigenol 3-*O*-*β*-D-xylopyranoside (**16**)^14^, 3-*O*-β-D-xylopyranosyl-24*S*,25-dihydroxy-15-oxo-acta-(16*R*,23*R*)-16,23-monoxoside (**17**)^18^, cimiricaside A(**18**)^19^, and 7,8-didehydro-25-anhydrocimigenol-3-*O*-*β*-D-xylopyranoside (**19**)^17^ (Figure 1).

### ChE assay

**Figure 2.**
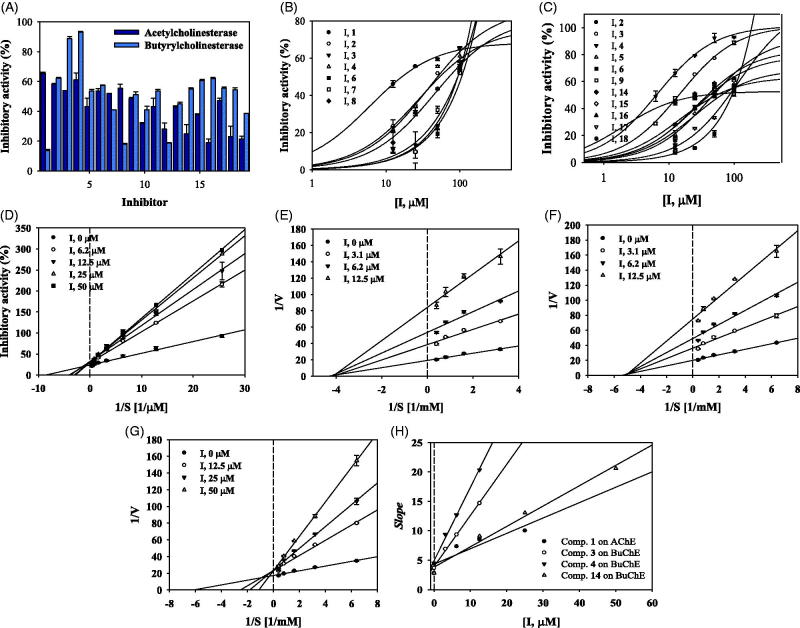
Inhibitory activity of compounds **1**–**19** at 100 μM on AChE and BuChE (A). IC_50_ values of them on AChE (B) and BuChE (C). Lineweaver-Burk plots of compound **1** on AChE (D) and of compounds **3**, **4**, and **14** on BuChE (E-G). Secondary plot of compounds **1**, **3**, **4** and **14** (H).

To screen for the ability of the isolated compounds **1**–**19** to block catalytic reaction of ChE, they were analysed *in vitro* at 100 μM concentration using a UV-spectrophotometer. As shown in Figure 2(A) and Table 1, compounds **1**–**4** and **6**–**8**, and compounds **2**–**6**, **9** and **14**–**18** exhibited over 50% inhibitory activity against AChE and BuChE, respectively. To calculate their IC_50_ values, these compounds were subjected to an enzyme assay at a variety of concentrations. They caused decreases in activities of the two ChEs, with gradual or sharp slopes in activity curves in a dose-dependent manner (Figure 2(B,C), Table 1). These results showed that compounds **1**– **4** and **6** – **8** had IC_50_ values ranging from 16.7 ± 1.9 to 95.8 ± 5.1 μM against AChE. In particular, compound **1** had an IC_50_ value of 16.7 ± 1.9 μM. Compounds **2**–**6**, **9** and **14**–**18** were revealed to have IC_50_ values ranging from 6.5 ± 2.5 to 90.9 ± 6.0 μM against BuChE. Compounds **3**, **4** and **14** exhibited greater inhibitory activity against BuChE than the other compounds tested, with IC_50_ values of 13.8 ± 1.2, 6.5 ± 2.5 and 22.6 ± 0.4 μM.

**Table 1.** Inhibitory activity of compounds **1**–**19** on two ChEs.

Compounds	AChE		BuChE	
	100 μM (%)	IC_50_ (μM)	100 μM (%)	IC_50_ (μM)
1	65.4 ± 0.9	16.7 ± 1.9	13.6 ± 0.7	N.T
2	58.0 ± 0.6	52.4 ± 3.7	62.3 ± 0.3	37.1 ± 1.5
3	53.8 ± 0.1	95.8 ± 5.1	88.7 ± 1.3	13.8 ± 1.2
4	61.0 ± 4.6	48.0 ± 6.8	93.1 ± 0.6	6.5 ± 2.5
5	43.1 ± 5.6	N.T	53.6 ± 0.9	60.9 ± 1.2
6	53.7 ± 1.6	94.9 ± 3.5	57.3 ± 0.3	90.9 ± 6.0
7	52.0 ± 0.1	92.1 ± 2.2	41.0 ± 0.1	N.T
8	55.4 ± 2.6	69.5 ± 1.2	18.0 ± 0.4	N.T
9	48.6 ± 0.6	N.T	51.0 ± 1.9	35.1 ± 1.2
10	31.8 ± 0.5	N.T	41.1 ± 2.2	N.T
11	15.8 ± 4.3	N.T	14.0 ± 2.6	N.T
12	28.0 ± 4.0	N.T	18.6 ± 0.3	N.T
13	42.8 ± 0.8	N.T	44.9 ± 1.1	N.T
14	24.9 ± 6.2	N.T	55.0 ± 0.7	22.6 ± 0.4
15	37.1 ± 0.5	N.T	60.7 ± 0.6	33.3 ± 5.0
16	19.0 ± 2.5	N.T	62.0 ± 0.8	31.0 ± 5.5
17	47.1 ± 1.6	N.T	55.4 ± 0.8	84.1 ± 5.7
18	23.1 ± 6.8	N.T	54.4 ± 1.3	51.0 ± 0.5
19	21.5 ± 1.7	N.T	38.5 ± 0.1	N.T
Tacrine^b^		0.123 ± 1.5		0.011 ± 0.4

N.T: not test.

^a^Compounds were tested three times.

^b^Positive control.

### Enzyme kinetics on AChE and BuChE

As indicated in Figure 2(D), compound **1** was competitive inhibitor which observed to have same *V*_max_ value, and different *K*_m_ values at 6.2–50 μM concentration on AChE. Compounds **3** and **4** were confirmed as noncompetitive mode due to various *V*_max_ values and a *K*_m_ value according to respective concentrations on BuChE (Figure 2(E,F)). Whereas, compound **14** was revealed to take the binding into activity site by competing with substrate (Figure 2(G)). Additionally, these results were calculated with *K*_i_ values of the potential inhibitors using secondary replot. Compound **1** was calculated to be 16.2 ± 0.9 μM on AChE. Compounds **3**, **4** and **14** were solved to be 4.9 ± 2.1, 3.5 ± 1.5 and 10.7 ± 1.3 μM on BuChE (Figure 2(H), Table 2).

**Table 2.** Enzyme kinetics of compounds **1**, **3**, **4** and **14** against two ChEs.

	Binding mode	*K*_i_ (μM)
1	Competitive type (AChE)	16.2 ± 0.9
3	Non-competitive type (BuChE)	4.9 ± 2.1
4	Non-competitive type (BuChE)	3.5 ± 1.5
14	Competitive type (BuChE)	10.7 ± 1.3

### Molecular docking of inhibitors with AChE and BuChE

**Figure 3.**
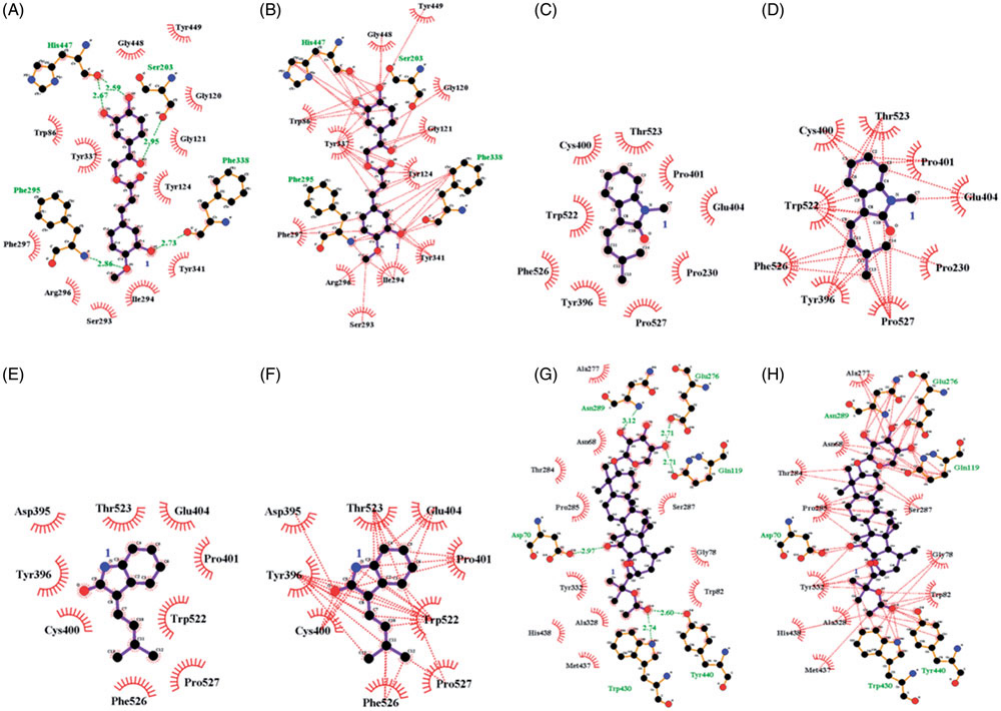
Hydrogen bonds (A) and hydrophobic interaction (B) between compound **1** and AChE. Hydrogen bonds and hydrophobic interaction between compounds **3** (C and D), **4** (E and F) and **14** (G and H) with BuChE.

These findings suggest that compounds **1**, **3**, **4** and **14** may bind with either AChE or BuChE. An inhibitor (**1**) was found to dock into the active site of AChE, thus acting as a competitive inhibitor of this enzyme. This inhibitor was fitted into the binding site in a stable position with an Autodock score of –9.42 kcal/mol. Compound **1** formed five hydrogen bonds (Ser203: 2.95 Å; Phe295: 2.86 Å; Phe338: 2.73 Å; His447: 2.59 Å and 2.67 Å) with four amino acids in AChE and had a hydrophobic interaction with amino acids surrounding the active site (Figure 3(A,B)). Compounds **3**, **4** and **14** exhibited molecular docking with BuChE. The noncompetitive inhibitors (**3** and **4**) were simulated in a blind docking test to search for possible binding with BuChE. The competitive inhibitor (**14**) docked into BuChE in the method described earlier. As shown in Figure 3(C–F), compounds **3** and **4** were confirmed to have hydrophobic interactions with amino acids, but not to form hydrogen bonds. The predicted binding site was proposed as the location where the inhibitor clustered with a low Autodock score. In particular, compound **4** was stably placed in the active site, with the top five positions scoring from –7.25 to 7.26 kcal/mol (Supplementary Figure S1). Enzyme kinetic results showed that compound **4** preferentially bound to the allosteric site. Therefore, the catalytic site was excluded as a binding site for this compound (**4**). Our results predicted the binding site as that with the lowest Autodock score for the next cluster, similar to the results for compound **3**. In addition, compound **14** exhibited hydrophobic interactions with seventeen residues and formed six hydrogen bonds (Asp70: 2.97Å; Glu119: 2.71 Å; Glu276: 2.71 Å; Asn289: 3.12 Å; Trp430: 2.74 Å; Tyr440: 2.60 Å) with six amino acids in the active site of BuChE (Figure 3(G,H)).

### MDs of inhibitors with AChE and BuChE

**Figure 4.**
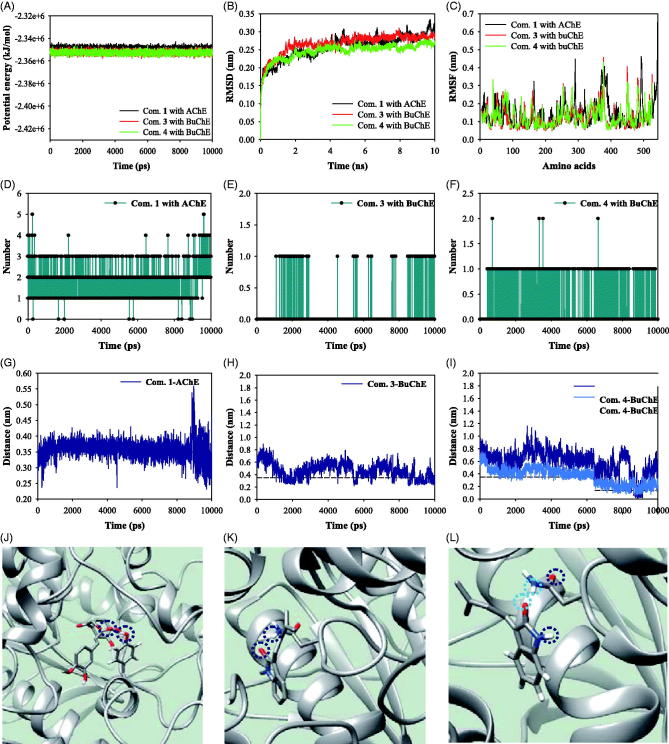
The potential energy (A), RMSD (B), RMSF (C), and hydrogen bonds (D–F) of respective compounds **1**, **3** and **4** with receptor for 10,000 ps. The distance (G–I) of respective compounds **1**, **3** and **4** with key amino acids (J–L).

We performed MD simulations to study the stability of the inhibitor-ChE complex in solution at 300 K under 1 bar of pressure. The complexes of AChE with compound **1**, and BuChE with compounds **3** and **4** were simulated stably with potential energies of about –2.35 × 10^6^ kJ/mol (Figure 4(A)). As indicated in Figure 4(B,C), each enzyme exhibited root mean square derivation (RMSD) values below 3.5 Å distance and root mean square fluctuations (RMSF) below 5.0 Å distance. Compound **1** formed about 1–3 hydrogen bonds with the active site of AChE for 10 ns (Figure 4(D)). Compounds **3** and **4** created 0–1 hydrogen bonds in the allosteric site of BuChE (Figure 4(E,F)). Furthermore, these results indicated the key amino acids interacting with inhibitors during MD simulation. Analysis of complex formation during a 1-ns interval of MD simulation showed that compound 1 participated in hydrogen bonding with Tyr337 in the active site of AChE (Figure 4(G,J)). Compounds **3** and **4** were located within 3.5 Å of Asn228 in the predicted allosteric site of BuChE (Figure 4(H,I,K,L)). Compound **1** maintained a 3.5 Å distance with Tyr337 for 10 ns. Compounds **3** and **4** occasionally approached Asn228 at a 3.5 Å distance.

## Conclusions

AD is a neurodegenerative disease caused by destruction of neurons in the central nervous system^20^. Cycloartane triterpenoids and cinnamic acid derivatives from the roots *of C. dahurica* have been reported to have a neuroprotective effect on PC12 cells^9^^,^^11^. The cholinergic hypothesis of AD is supported by increased memory and cognition function after binding of ACh to ACh receptors in the brain^8^^,^^21^. Therefore, AChE and BuChE are considered promising target enzymes for treating AD disease due to their effect of decreasing ACh levels^4^^,^^8^.

Our study led to isolation of cinnamic acids (**1** and **2**), indolinones (**3** and **4**), and triterpenoid derivatives (**5**–**19**) from the roots of *C. dahurica*. We analysed these compounds to evaluate their inhibitory activity on both AChE and BuChE. Compound **1** has an IC_50_ value of 16.7 ± 1.9 μM against AChE, while compounds **3** and **4** were determined to have IC_50_ values of 13.8 ± 1.2 and 6.5 ± 2.5 μM against BuChE, respectively. According to BuChE assay results, triterpenoid glycosides showed more potent inhibitory activities than those of their aglycones except for compound **19**. Above all, indolinone derivatives (**3** and **4**) were highly potential inhibitors compared to the others. In reported studies, alkaloid derivatives, such as atherosperminine, (+)-*N*-methylisococlaurine, berberine, 9-amino-1,2,3,4-tetrahydro acridine, and rivastigmine, were found to be famous ChEs inhibitor^12^^,^^20^^,^^21^. Moreover, blood brain barrier (BBB) plays a role to keep neuronal cells from neurotoxic substances of outside. However, BBB transporters of glucose, phenylalanine, araginine and lactate are responsible for transporting small molecules, such as deoxyglucose, galactose, lysine, pyruvate and guanosine, into brain^22^^,^^23^. Especially, compound **4** having 199 Da alkaloid may overcome the block of BBB and potentially invade into brain.

Based on the enzyme kinetic study, compound **1** was shown to block catalytic reaction by interacting with the active site of AChE. Compounds **3** and **4** were revealed to have affinity for the allosteric site of BuChE. Their binding positions were predicted for the active or allosteric sites using the Autodock 4.2 package. Moreover, MD analysis led us to propose the key amino acid involved in ligand-receptor interactions. As a result, the ketone form of the ester in cimiciphenone (**1**) exhibited hydrogen bonding with the aromatic hydroxyl group of Tyr337 in the active site of AChE during simulation. To develop a new cinnamic acid moiety of AChE, chemists should consider compounds that are capable of interaction with Tyr337. (*E*)-3–(3′-methyl-2′-butenylidene)-1-methyl-2-indolinone (**3**) and (*E*)-3–(3′-methyl-2′-butenylid-ene)-2-indolinone (**4**) participated in hydrogen bonding with Asn228 located at its predicted binding site on BuChE. It is necessary to develop a prenyl indolinone derivative as a noncompetitive inhibitor to enhance hydrogen bonding with this polar amino acid (Asn228). In our research, we identified the key amino acids, which could not be found through molecular docking, using MD analysis. Among cycloartane triterpenoids and cinnamic acid derivatives that show neuroprotective activity^9^^,^^11^, cimiciphenone (**1**), (*E*)-3–(3′-methyl-2′-butenylidene)-1-methyl-2-indolinone (**3**) and (*E*)-3–(3′-methyl-2′-butenylidene)-2-indolinone (**4**) showed promise as potential inhibitors of AChE and BuChE, respectively. Compound **1** was determined to be the optimal compound for development as a competitive inhibitor of AChE, while compounds **3** and **4** may provide a new skeleton for noncompetitive inhibitors of BuChE.

## Supplementary Material

Supplemental Material
