# Supplementary material for: The insight of in vitro and in silico studies on cholinesterase inhibitors from the roots of Cimicifuga dahurica (Turcz.) Maxim
Source: J Enzyme Inhib Med Chem. 2018 Oct 5;33(1):1174–80. doi: 10.1080/14756366.2018.1491847 (PMC6179041; doi:10.1080/14756366.2018.1491847)
Supplement: Supplemental Material [file IENZ_A_1491847_SM1327.pdf]

SUPPORTING INFORMATION

The insight of *in vitro* and *in silico* studieson cholinesterase inhibitors from the roots of *Cimicifugadahurica*(Turcz.) Maxim.

Jang Hoon Kim,[a,#] Nguyen Phuong Thao,[b,#] Yoo Kyong Han, [d]Young Suk Lee, [d] Bui Thi Thuy Luyen,[c] Ha Van Oanh, Young Ho Kim[d,]*, and Seo Young Yang[d,]*

[a]*Advanced Radiation Technology Institute, Korea Atomic Energy Research Institute, Jeoungeup, Jeollabuk-do 56212, Republic of Korea*

[b] *Institute of Marine Biochemistry (IMBC), Vietnam Academy of Science and Technology (VAST), 18-Hoang Quoc Viet, Hanoi, Vietnam*

[c] *Hanoi University of Pharmacy, 13–15 Le Thanh Tong, Hanoi, Vietnam*

[d]*College of Pharmacy, Chungnam National University, Daejeon 34134, Republic of Korea*

[#] These authors contributed equally to this work

*Corresponding authors:

Seo Young Yang Ph.D.

College of Pharmacy, Chungnam National University, Daejeon 34134, Republic of Korea

Tel: 82-42-821-7321 Fax: 82-42-823-6566 E-mail: syyang@cnu.ac.kr

Young Ho Kim Ph.D.

College of Pharmacy, Chungnam National University, Daejeon 34134, Republic of Korea

Tel: 82-42-821-5933 Fax: 82-42-823-6566 E-mail: yhk@cnu.ac.kr

B

A

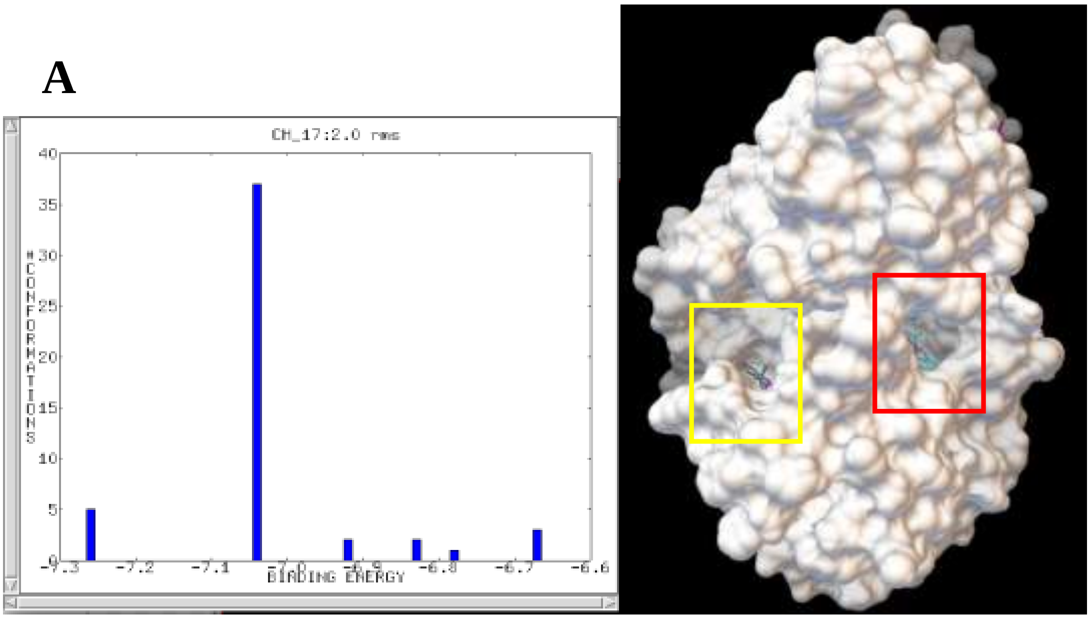

Figure S1. Cluster and Audodock score of compound **4** with BuChE (A). Predicted docking pose of compound **4** with BuChE (B) (red box: activity site; yellow box: predicted binding site).

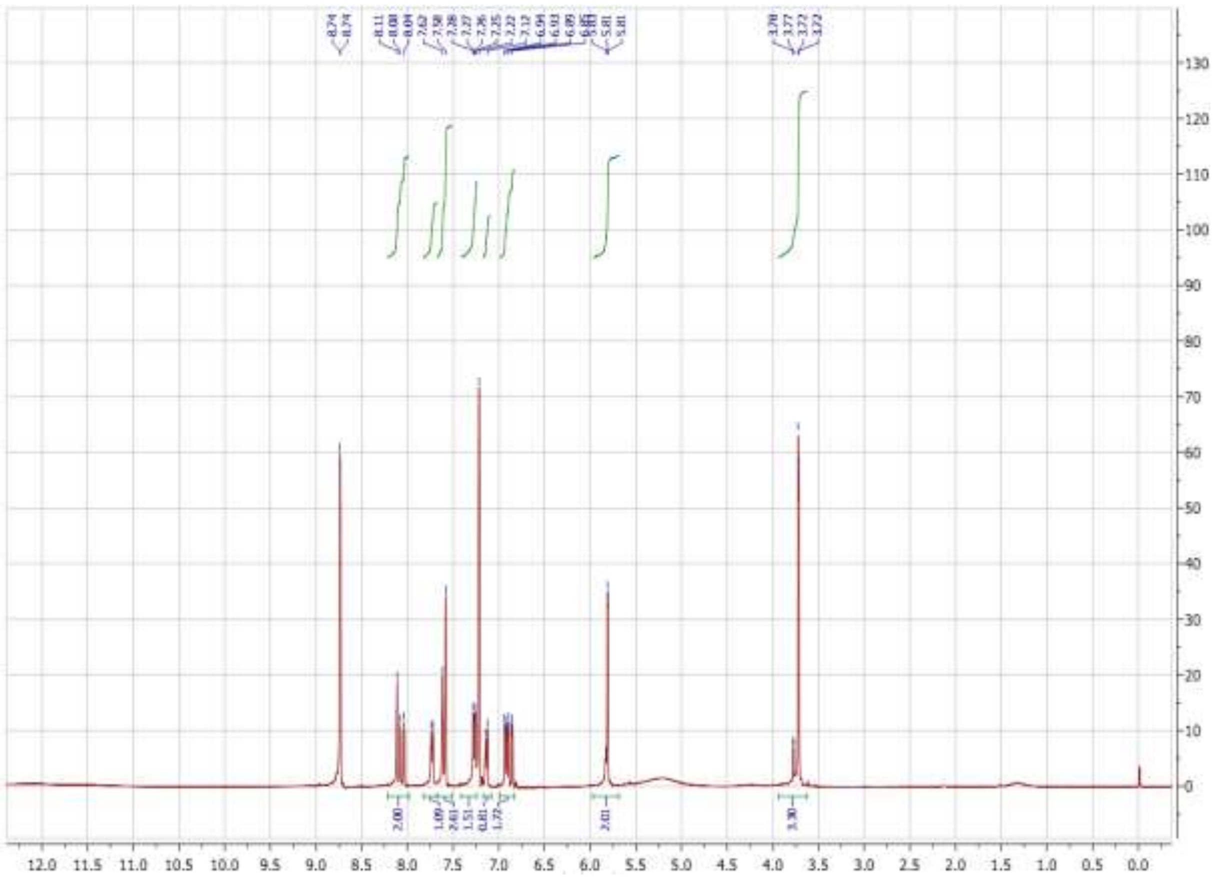

Figure S2. ¹H NMR spectrum (Pyridine-d_5, 500 MHz) of compound **1**.

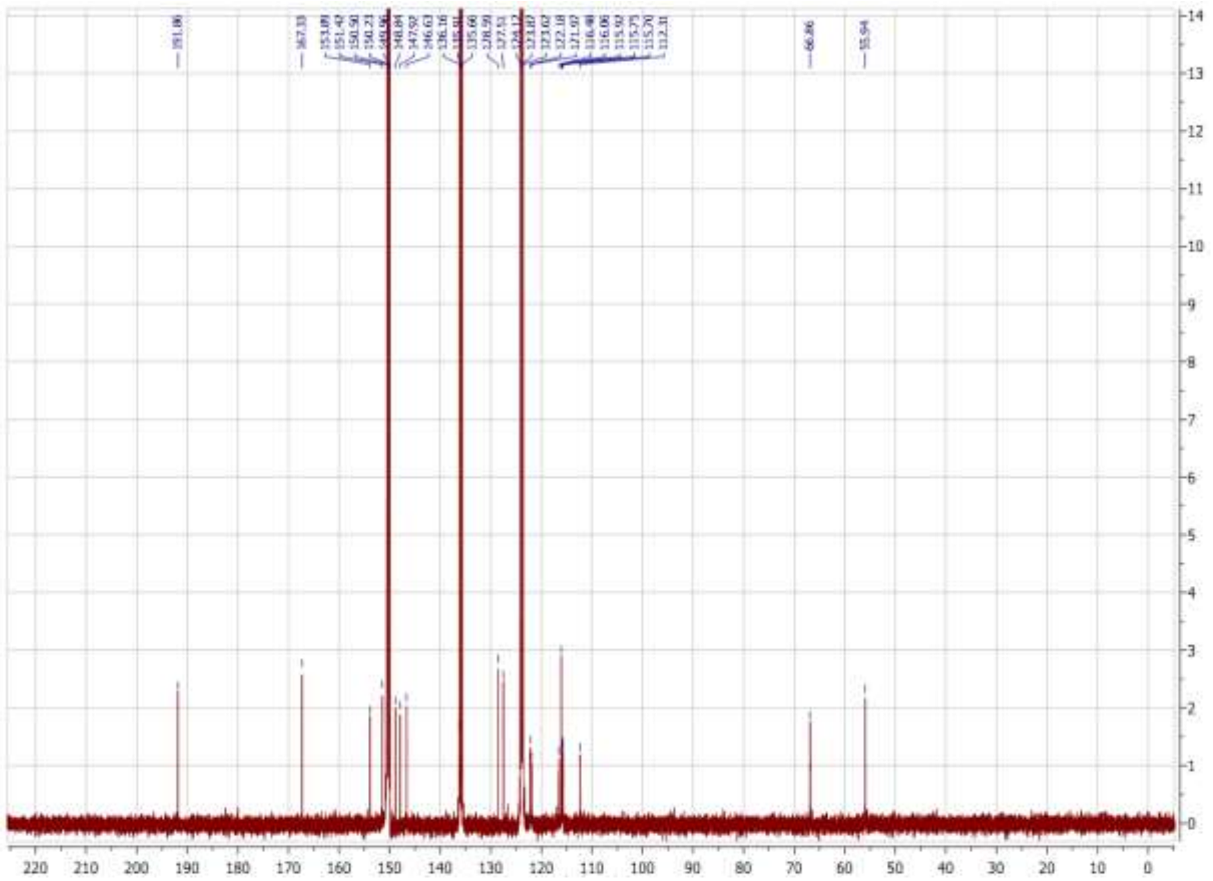

Figure S3. ¹³C NMR spectrum (Pyridine-d_5, 500 MHz) of compound **1**.

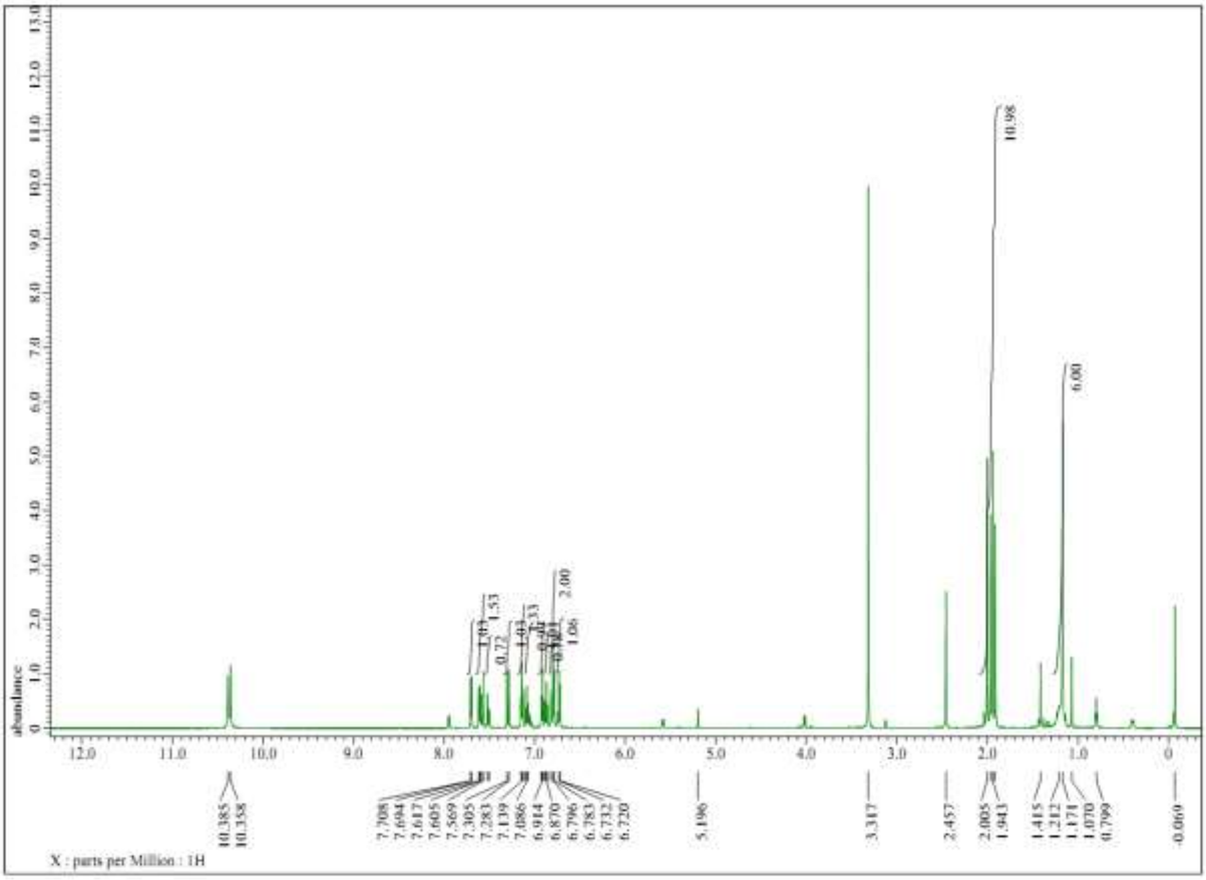

Figure S4. ^{1}H NMR spectrum (DMSO-d_6, 500 MHz) of compound **3**.

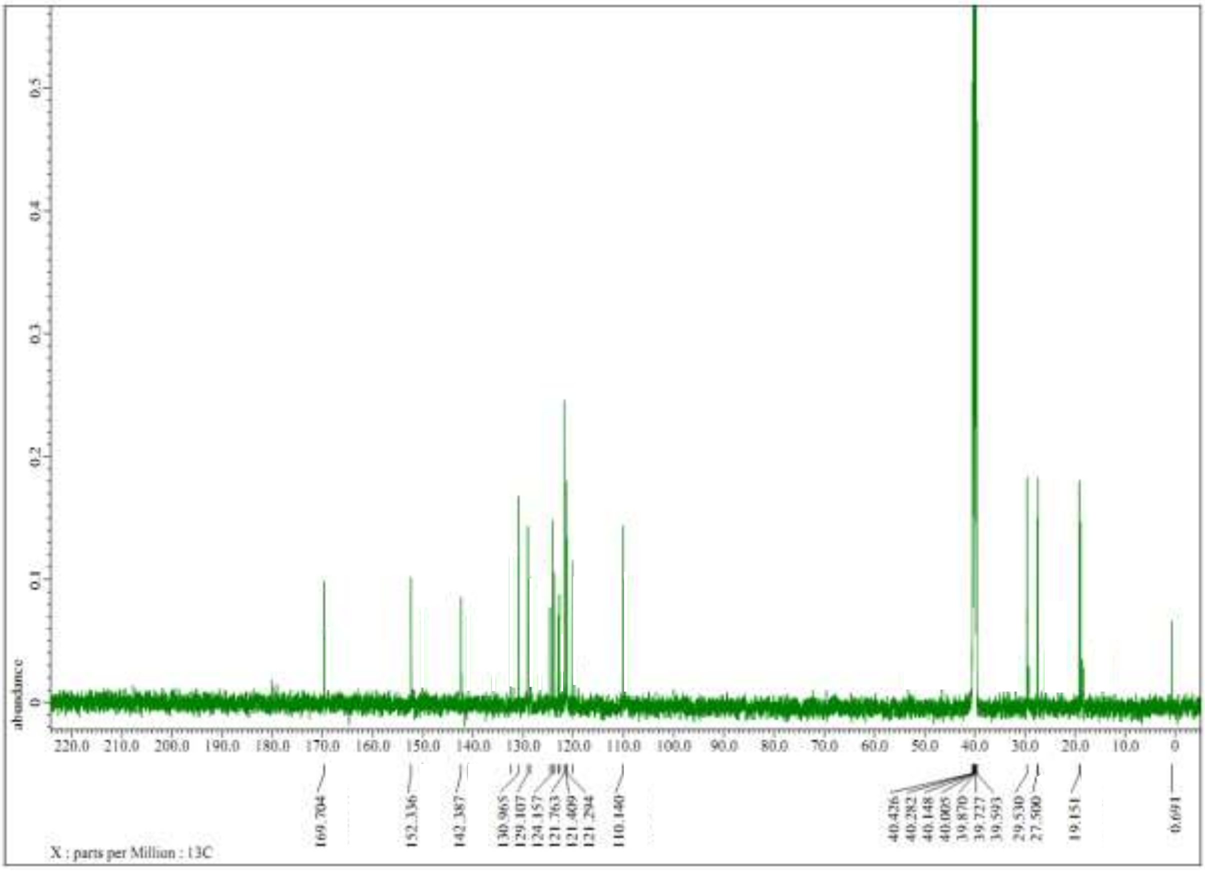

Figure S5. ^{13}C NMR spectrum (DMSO-d_6, 500 MHz) of compound **3**.

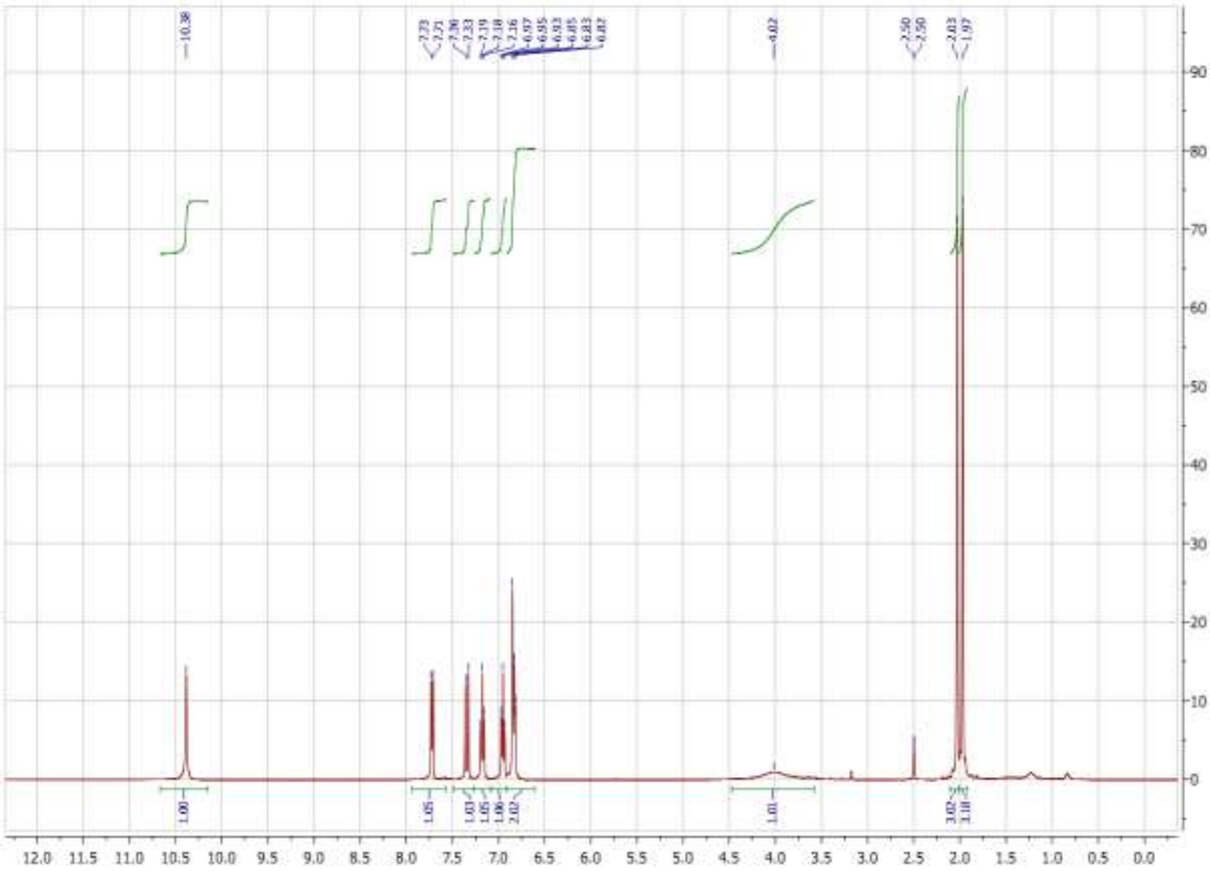

Figure S6. ^{1}H NMR spectrum (DMSO-d_6, 500 MHz) of compound **4**.

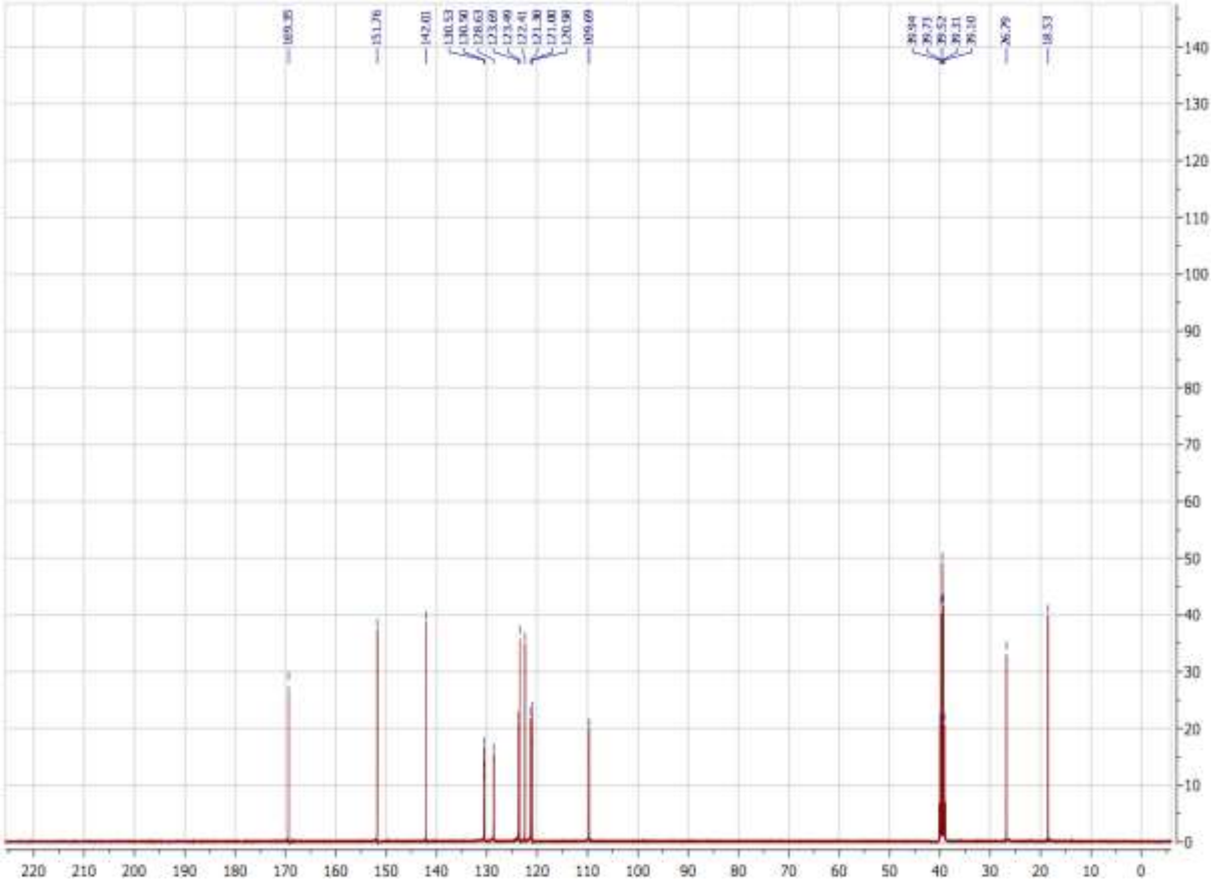

Figure S7. ^{13}C NMR spectrum (DMSO-d_6, 500 MHz) of compound **4**.

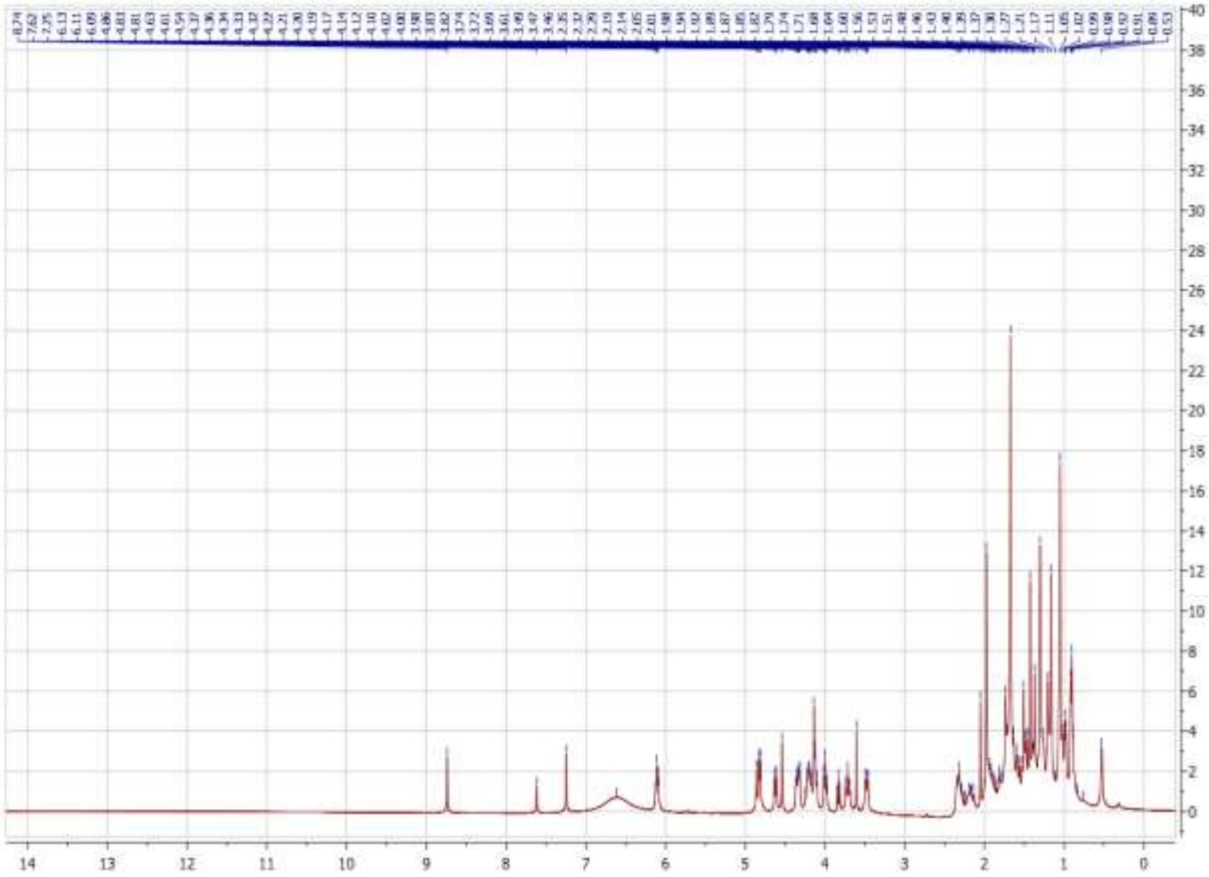

Figure S8. ^{1}H NMR spectrum (Pyridine-d_5, 500 MHz) of compound **14**.

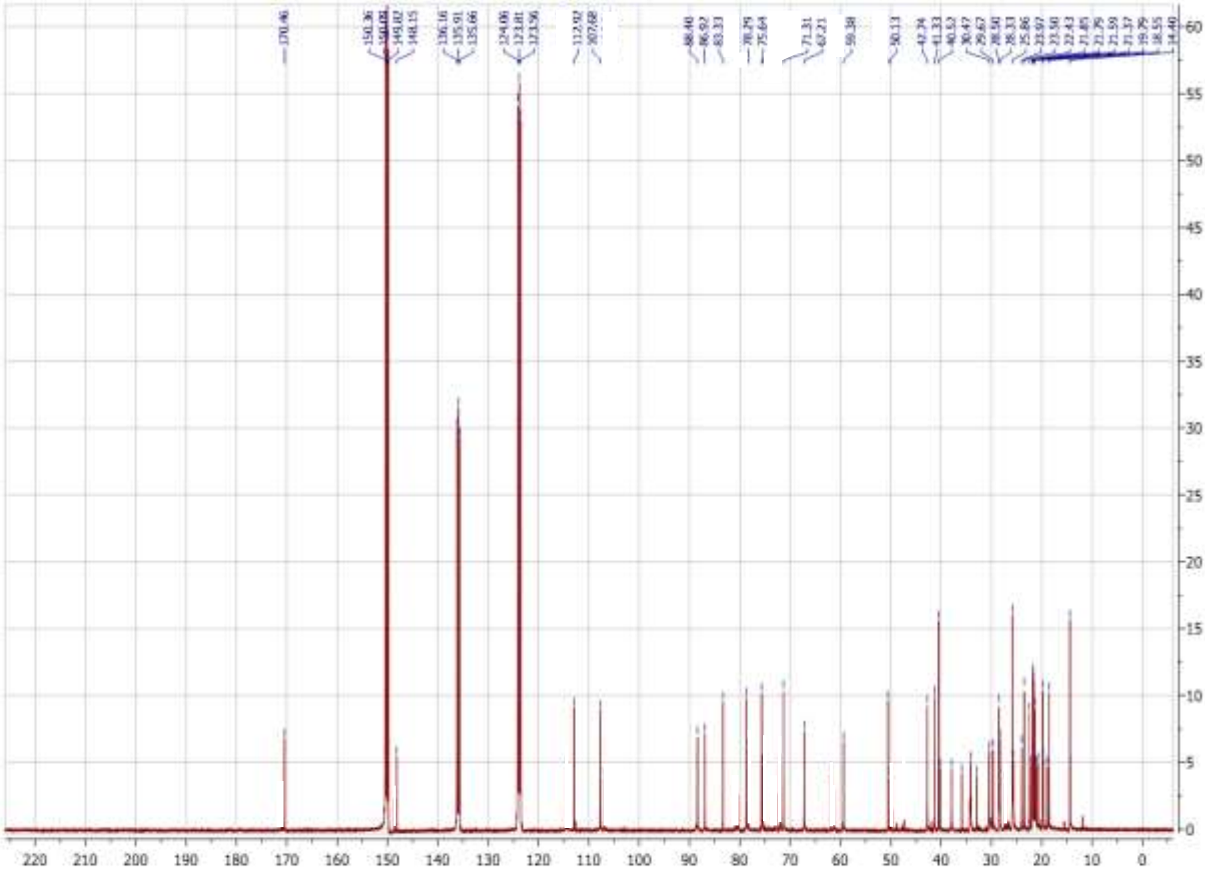

Figure S9. ^{13}C NMR spectrum (Pyridine-d_5, 500 MHz) of compound **14**.
